# Condom literacy, self-efficacy, and HIV-risk behaviors: a cross-sectional analysis of adolescent girls in northwestern Botswana

**DOI:** 10.3389/fpubh.2026.1830061

**Published:** 2026-06-19

**Authors:** Francis Barchi, Helen Apps, Peggie Ramaphane

**Affiliations:** 1 Edward J. Bloustein School of Planning & Public Policy, Rutgers University, New Brunswick, NJ, United States; 2 WoMen Against Rape, Maun, Botswana

**Keywords:** adolescent girls, AGYW, condoms, HIV-risk behaviors, self-efficacy, sub-Saharan Africa

## Abstract

**Introduction:**

Despite global reductions in HIV incidence, adolescent girls in Sub-Saharan Africa remain disproportionately burdened.

**Methods:**

This study used cross-sectional data collected from 306 school-going girls ages 14–19 in northwestern Botswana in 2021 to identify behavioral and attitudinal factors associated with knowledge of correct condom use and the intention to use condoms consistently. Data were collected through self-administered, tablet-based surveys containing questions relating to HIV and condom knowledge, condom use, and relationship norms. All data were self-reported. Bivariate and multivariate analyses examined correlations with two outcome variables: reported knowledge of correct condom use and perceived ability to insist on condoms in every sexual encounter.

**Results:**

Most participants were 15–16 years old. Only 15% reported having had sexual intercourse. Few reported engaging in activities generally associated with HIV-risk behaviors, such as smoking (1.3%), alcohol use (7.2%), or substance use (4.6%). Girls who reported they had not had sexual intercourse or who did not feel they could insist on condom use had 70 and 80% lower odds, respectively, of reporting they knew how to use condoms correctly compared to more experienced or confident peers. Girls who knew there was no cure for AIDS had 2.7 times greater odds of reporting they knew how to use condoms correctly. Participants who were unaware of their HIV status had approximately 80% lower odds of reporting they could insist on condoms compared to their status-aware peers. Knowledge of correct condom use and attitudes about relationships were not significant correlates of perceived ability to insist on condoms when controlling for other covariates. Several indicators of condom-use self-efficacy, including confidence in suggesting condoms or refusing condom-less sex and communication with one’s sexual partner were significant correlates of perceived ability to insist on condom use.

**Discussion:**

Most understanding of factors contributing to adolescent HIV-risk behaviors is based on studies of males and females combined or older, more sexually active young adults. Results from this study, which focused exclusively on adolescent girls, the majority of whom were not yet sexually active, point to potential targets of opportunity to reduce the likelihood of unprotected sexual initiation in this particularly vulnerable group.

## 1 Introduction

Globally, 1.3 million people became newly infected with HIV in 2024 (1). While new cases of HIV continue to decline overall, adolescents remain disproportionately burdened, accounting for 27% of new cases despite representing only 16% of the global population (2). Among this group, seven of every ten new cases of HIV among adolescents ages 15–19 years occurred in girls (3). Of the 4,000 adolescent girls and young women (AGYW) newly infected with HIV in 2024, 82.5% occurred in Sub-Saharan Africa.

Condoms figure prominently in HIV-prevention efforts. When used consistently and correctly, condoms are highly effective in preventing the transmission of HIV and other sexually transmitted infections (4). Despite their safety, effectiveness, and widespread availability, however, condom use among adolescents and young people in Sub-Saharan Africa, frequently measured as a percentage of those who used a condom at the time of their most recent sexual intercourse, is low (5, 6).

A significant body of research has examined the factors that drive the underutilization of condoms among adolescents and young adults, including biological factors such as age (6) and being female (7). Among the reported behavioral factors contributing to non-use of condoms is alcohol consumption (7, 8) and high-risk sexual behaviors including early sexual debut (6, 7, 9–12), multiple sexual partners (6, 7), and sexual self-efficacy (13). Patriarchal norms that emphasize women’s inferiority in sexual matters and decision-making, endorsement of prevailing gender norms, and perceived acceptability of sex have been found to be predictive of condom use by adolescents and young adults (14–17). Misperceptions around condoms can also result in non-use or inconsistent use of condoms (15, 16, 18).

Since Albert Bandura’s influential 1991 article positing that self-efficacy might be an effective tool to use in HIV interventions (19), numerous researchers have endeavored to measure the role that self-efficacy plays in condom use. Defined by Bandura as “people’s beliefs about their capabilities to exercise control over their own level of functioning and over events that influence their lives” (p. 118) (20), self-efficacy has since been shown to be predictive of sexual risk-taking, and condom use (5, 21–24).

Adolescents remain a population of key concern in HIV prevention in Botswana, with adolescents making up 18.7% of the population in 2024 yet accounting for 27% of all new HIV cases in the country (25). Studies examining the factors associated with consistent and correct condom use within this age cohort have been limited and vary considerably in sample makeup, age-range, setting, and methodological approaches used. Those studies examining perceived intention to use condoms or consistent condom use have included both males and females.

Despite the widely acknowledged significance of gender and age in HIV-risk behaviors and vulnerability to infection, few studies in Botswana and elsewhere in Sub-Saharan Africa have focused exclusively on adolescent girls ages 15–19 years of age. ‘Gender’ has featured predominantly as an independent variable in analyses examining both male and female respondents, potentially causing researchers to either over- or under-attribute the extent to which attributes such as age, HIV- and condom-knowledge, gender norms, and sexual behaviors may be significant correlates of adolescent girls’ perceived ability to consistently and correctly use condoms. Additionally, the majority of studies have focused on the knowledge, attitudes, and behaviors of those adolescents and young adults who report having had sexual intercourse. Although reported age of first sex in many studies suggests that many adolescents in southern Africa, particularly younger ones, have not yet had sexual intercourse (26–28), the perceptions and intentions of this group regarding HIV, condom use, and relationships have garnered far less attention.

This study begins to address these gaps by its exclusive focus on adolescent girls and the factors associated with their perceived knowledge of correct condom use and intentions to insist on sex with condoms. The study aims to identify areas where adolescent girls may lack accurate information and/or confidence around condom use and negotiation. Such areas may be opportunities for capacity-building and behavioral interventions that contribute to safer sex practices among girls already having sexual intercourse and promote condom use at first sex by those as yet sexually uninitiated. We hypothesized that girls who responded that they had had sexual intercourse would report greater confidence in their knowledge of correct condom use and their intentions to insist on sex with condoms than girls who reported that they had not yet had sexual intercourse.

## 2 Materials and methods

This analysis used cross-sectional data collected in June–December 2021 as part of a multi-phase, multi-year study of adolescent sexual and reproductive health in rural northwestern Botswana. The study was undertaken by a collaborative partnership including staff members at WoMen Against Rape (WAR), a human rights non-governmental organization based in Maun, Botswana, and researchers at Rutgers University. WAR provides counselling and support services for victim-survivors of gender-based violence and offers sexual and reproductive health rights (SRHR) programming for adolescents attending secondary school.

### 2.1 Study sample

The Northwest District is home to approximately 165,000 residents. At the time of this study, approximately 14,300 adolescents were attending secondary schools in the Northwest District. The adolescents included in this analysis were drawn from a convenience sample of 11 schools in the Northwest District of Botswana identified in consultation with the regional office of the Botswana Ministry of Basic Education and representing the majority of the government secondary schools in the district. In each school, the study team recruited students attending one of the open periods during which life-skills and comprehensive sexuality education are generally taught. Approximately 50–60 students ranging in ages and class level attend such sessions in each school, resulting in a target population of 550–660 students. Students under the age of 14, those whose parents had declined to have their child participate, and those students who themselves did not wish to participate, were excluded. Among the 594 boys and girls who were invited to participate in the parent study, 21 students refused. Of those who consented, three were found to be underage and their responses were dropped from the sample. An additional 13 students who did not identify their gender were also dropped from the sample. Four students identifying their gender as ‘other’ were also excluded due to concerns that this designation, combined with other demographic details, posed a risk of identification. These exclusions resulted in an analytic sample of 553 adolescent males and females. The current analysis was limited to responses from the 306 female participants in the parent study only.

### 2.2 Survey administration

Respondents self-administered surveys on tablets using Qualtrics survey software.^1^ Computerized self-administered questionnaires (CSAQ) are a particularly useful tool in situations such as this where students are being asked questions that are sensitive in nature; CSAQs reduce the chance of interviewer bias, enhance privacy, and allow the use of complex skip patterns in questions that might otherwise be too confusing (29). The structured survey questions and response choices were written in both English and Setswana, allowing respondents to use whichever of the two languages they found most comfortable. Questions were initially written in English and then translated into Setswana, the local language, by a native speaker, and subsequently back translated into English by a member of the study team who was also a native speaker. The majority of questions were pilot tested in a sample of 27 students attending private school in Maun, the district seat, and adjusted for clarity and readability as needed. The study team received approval from school administrators and the responsible ethics committees to use a “passive” consent process for parental permission that gave parents the opportunity to opt-out of the survey on behalf of their children. On the day of the survey, a member of the research team explained the purposes of the survey, the use of the data, the risks and benefits of participation, and students’ rights as study participants. Students indicated their assent/consent to participate by ticking a required box in the survey before accessing the study questions. They were advised that they did not have to answer any questions that made them uncomfortable or they found too sensitive. Students were encouraged to speak with a WAR counselor if any questions reminded them of unsettling experiences. No names or personal health information were collected.

### 2.3 Measures

The questions used in this study were adapted for use in this setting from the World Health Organization’s Illustrative Questionnaire for Interview Surveys with Young People, the 2005 Botswana Global School-Based Student Health Survey (GSHS), and the Condom-Use Self-Efficacy Scale (30–33). Key outcome variables of interest in this analysis were based on girls’ responding “agree,” “disagree” or “I do not know” to each of two statements: “I feel I know how to use a condom correctly.” and “I feel I can insist on condoms every time I have sexual intercourse.”

The survey included 115 questions presented in 11 sections; these included demographics (19 questions), alcohol and substance use (11 questions), sexual behaviors (11 questions), sources of health information (5 questions), knowledge and use of contraceptives (14 questions), HIV and other STIs (9 questions), condom use (16 questions), views on relationships (19 questions), and health service utilization (7 questions). Demographic, attitudinal, and behavioral measures from the survey data were used to examine their association with a respondent’s reported knowledge of correct condom use and ability to insist on condom use. Demographic measures included grade level, age, substance use, and sexual experience. Girls who indicated that they had had sexual intercourse at least once in their lifetimes were asked seven questions about their sexual behaviors and condom use.

All respondents were asked if they had ever been tested for HIV, if they knew their HIV status and if they were aware of other diseases that can be transmitted through sexual intercourse. Household-level variables included questions about the education level of the eldest female in a respondent’s home, the gender of the head of their household and whether they had lived with persons other than a parent in the past 12 months. As a proxy for poverty, a ‘wealth index’, reported in quintiles, was created from responses indicating if anyone in the household owned various tangible assets including land, house, livestock, radio, television, cell phone, canoe/boats, donkey cart, bicycle, or car/truck. Respondents were also asked a series of questions about their access to food, sources of household access to water, sanitation, and electricity. Additional questions assessed respondent knowledge about HIV/AIDS, their knowledge about condoms and their self-efficacy with respect to condom use, as well as their attitudes regarding various normative statements about gender.

### 2.4 Analysis strategy

All data were analyzed using Stata statistical software version 16 (34). Descriptive statistics were computed for all variables, and bivariate analyses were run to examine their correlation with the two outcome variables of interest, *able to use a condom correctly* (correctly) and *able to insist on condom use every time* (insist). All three possible levels of responses (agree, disagree, do not know) were included in the univariate and bivariate analyses. Missing observations were excluded in the Pearson’s chi-square statistics, and Fisher’s exact tests were used in contingency tables containing cell counts less than five. Correlations between sexual behaviors and condom use among sexually experienced respondents and the outcome variables were not computed given the small number of respondents (*n* = 46).

Logistic regression models were created to examine the association between respondent attributes, knowledge, and attitudes and each of the outcome variables. At the multivariate level, responses for the outcome variables *correctly* and *insist* were collapsed into two-levels (agree, disagree/do not know). Independent variables that had *p*-values of 0.05 or less in the relevant bivariate analysis were included in the appropriate multivariate model. Tests were run on each logistic regression model to see if the data met the assumption of collinearity. In both models, VIF values were all less than 1.6 and tolerance ranged from 0.63 to 0.97, indicating moderate correlation between variables. While several variables in each model may reflect facets of a girl’s underlying confidence and perceived ability, they were retained, as variance inflation factors and tolerance for them fell well below the threshold indicating severe multicollinearity (35).

List-wise deletion was used in the logistic regression models to eliminate those respondents with missing responses on any of the variables of interest to avoid inferential error (36). Only one variable, *gender sub-district location*, contained no missing values, and, as most responses were categorical, multiple imputation of missing values was impracticable. Sensitivity tests revealed no change in the directionality of the findings despite the loss in observations. An *a priori* power analysis was conducted using G*Power version 3.19.7 (37) to determine the minimum sample size required to test the study hypothesis. Results indicated the required sample to achieve a moderate effect size of 0.15 at a probability level of 0.05 was *N* = 149 for the multiple regression analyses. Thus, the obtained sample sizes of 244 and 236 for the models *correctly* and *insist*, respectively, were more than adequate to test the study hypotheses.

### 2.5 Ethics statement

The study was reviewed and approved by the institutional review boards at Rutgers University, the University of Botswana, the Health Research and Development Committee at the Botswana Ministry of Health and Wellness, and the Botswana Ministry of Basic Education. All students participating in this study did so with parental permission. All students provided assent/consent.

## 3 Results

### 3.1 Univariate analysis

This analysis is based on responses from 306 female secondary school students ranging in age from 14 to 19 years, the majority of whom were aged 15–16 years and enrolled in Forms 2 and 3 (junior secondary school). Very few respondents engaged in activities generally associated with adolescent risk behavior, including smoking cigarettes (1.3%), alcohol use (7.2%), and drug/substance use (4.6%). Thirty-two percent of these AGYW reported they had been tested for HIV, and approximately 28% reported knowing their HIV status (see Table 1). Most respondents (84%) lived in households with at least one parent, with only 16% reporting that they had not resided in a household with a parent at some point in the 12 months prior to the survey. Only 46 respondents (15%) reported having had sexual intercourse. Distribution of these respondents according to sexual risk behaviors and condom use is reported in Table 2.

**Table 1:** Distribution of respondents by sociodemographic and household attributes according to knowledge of correct condom use and reported ability to insist of condom use (*N* = 306).

Measure	Value	*n*	%	By correctly				By insist			
				No	Yes	*X* ^2^	*p*	No	Yes	*X* ^2^	*p*
Grade-level in secondary school	Form 1	43	14.1	29	11	3.56	0.470	18	22	22.35	<0.001
	Form 2	117	38.2	67	45			29	87		
	Form 3	81	26.5	48	31			9	70		
	Form 4	48	15.7	34	14			6	42		
	Form 5	12	3.9	7	4			1	11		
	Missing	5	1.6								
Age of respondent	14	30	9.8	20	9	2.36	0.797	8	22	9.53	0.090
	15	109	35.6	71	35			28	81		
	16	84	27.5	48	34			10	72		
	17	36	11.8	23	13			7	29		
	18	9	2.9	5	4			0	9		
	19	4	1.3	2	2			0	4		
	Missing	34	11.1								
Do you smoke cigarettes?	No	299	97.7	186	104	0.34	0.621*	61	233	1.04	0.585*
	Yes	4	1.3	2	2			0	4		
	Missing	3	1.0								
Do you drink alcohol?	No	280	91.5	179	94	3.43	0.064	60	218	3.15	0.088*
	Yes	22	7.2	9	11			1	19		
	Missing	4	1.3								
Have you ever taken drugs?	No	290	94.8	181	101	0.57	0.448	61	226	0.26	1.000*
	Yes	14	4.6	7	6			2	11		
	Missing	2	0.7								
Have you ever had sexual intercourse?	No	257	84.0	168	19	10.50	0.001	55	199	0.35	0.557
	Yes	46	15.0	81	26			8	37		
	Missing	3	1.0								
Highest level of education attained by the oldest female in the household	Primary or less	81	26.5	51	29	0.40	0.941	24	57	6.81	0.078
	Some secondary	31	10.1	19	9			7	22		
	Completed secondary	109	35.6	67	41			21	87		
	University or higher	85	27.8	52	28			11	71		
Head of household	Male head of household	196	64.1	114	75	3.04	0.081	39	156	0.00	0.967
	Female head of household	100	32.7	70	29			20	79		
	Missing	10	3.2								
In the past 12 months, has respondent ever lived without parents	No	255	83.3	154	91	0.25	0.616	46	204	5.45	0.020
	Yes	48	15.7	32	16			16	32		
	Missing	3	1.0								
Wealth index expressed in quintiles based on household ownership of land, House, livestock, radio, television, cell phone, canoe/boat, donkey cart, bicycle, car/truck	1st quintile	58	19.0	29	28	6.34	0.175	14	43	2.52	0.642
	2nd quintile	61	19.9	40	18			15	46		
	3rd quintile	56	18.3	38	18			9	47		
	4th quintile	70	22.9	46	24			12	58		
	5th quintile	44	14.4	30	12			8	36		
		17	5.6								
Frequency of not having food	Frequently/always	21	6.9	14	6	0.40	0.526	4	16	0.00	0.987
	Never/sometimes	276	90.2	168	99			16	218		
	Missing	9	2.9								
Water source for household	River, stream, rainwater or other	25	8.2	14	11	0.68	0.410	7	9	5.27	0.022
	Communal/private wells or taps	274	89.5	171	95			9	228		
	Missing	7	2.3								
Does household have electricity	No	109	35.6	61	45	2.82	0.093	26	36	0.84	0.360
	Yes	187	62.1	124	60			83	150		
	Missing	20	3.3								
Type of sanitation	Bush/other	69	22.6	37	30	2.48	0.116	15	54	0.07	0.791
	Private/communal pit latrine/flush toilet	230	75.2	148	77			46	181		
	Missing	7	2.3								

*Fisher’s exact test.

**Table 2:** Distribution of respondents by sexual behavior and condom use (*n* = 46).

Measure	Value	*n*	%
Have you ever had sexual intercourse?	No	257	84.0
	Yes	46	15.0
	Missing	3	1.0
If sex ever: How old were you the first time you had sexual intercourse?	11 years or younger	5	10.9
	12–14 years	9	19.6
	15–17 years	26	56.5
	over 17 years	5	10.9
	Missing	1	2.2
If sex ever: In the past 12 months, how many sexual partners have you had?	No sex in the past 12 months	11	23.9
	One person	24	52.2
	Two persons	6	13.0
	3–5 persons	3	6.5
	Missing	2	4.4
If sex ever: During the past 12 months, how frequently did you and your partner/s use condoms?	Never or rarely	7	15.2
	Sometimes	5	10.9
	Most of the time or always	22	47.8
	Missing	12	26.1
If sex ever: The first time you had sex, did you and your partner use a condom?	No	7	15.2
	Yes	39	84.8
If sex ever: The most recent time you had sex, did you and your partner use a condom?	No	6	13.0
	Yes	38	82.6
	Missing	2	4.4
If sex ever: Have you ever been forced to have sexual intercourse?	No	33	71.7
	Yes	13	28.3
If sex ever: Have you ever paid money or given gifts in exchange for sex?	No	39	84.8
	Yes	6	13.0
	Missing	1	2.2
If sex ever: Have you ever accepted money or gifts in exchange for sex?	No	40	87.0
	Yes	6	13.0
If sex ever: Has your family ever forced you to have sex with someone when you did not wish to?	No	43	93.5
	Yes	3	6.5

### 3.2 Bivariate analysis

Bivariate analyses for all variables used in this analysis and their association with the outcome variables of interest, *correctly* and *insist*, are reported for sociodemographic and household-level variables (Table 1), HIV knowledge (Table 3), and condom knowledge and self-efficacy (Table 4). Sexual experience, HIV testing, awareness of whether AIDS could be cured, and condom knowledge and self-efficacy were associated with a respondent reporting that she knew how to use a condom correctly. A respondent’s grade level in school, awareness of HIV status, having lived in a household without a parent at some time in the past 12 months, beliefs about HIV, and condom knowledge and self-efficacy were associated with a respondent’s reporting that she felt able insist on condom use every time. Age was significantly correlated with sexual experience at the bivariate level *X^2^*(5, *N* = 46) = 20.08, *p* = 0.001. Just over 67% of the adolescents who had had sexual intercourse did so for the first time between the ages of 15 and 17 years *X^2^*(15, *N* = 46) = 55.66, *p* < 0.001 (Table 5).

**Table 3:** Distribution of respondents by HIV knowledge according to reported knowledge of correct condom use and perceived ability to insist on condom use (*N* = 306).

Measure	Value	*n*	%	By correctly				By insist			
				No	Yes	X^2^	*p*	No	Yes	X^2^	*p*
It is possible to cure AIDS.	No	233	76.1	154	77	3.98	0.046	38	194	13.17	<0.001
	Yes	71	23.2	34	30			25	43		
	Missing	2	0.7								
A person with HIV always looks emaciated or unhealthy in some way.	No	150	49.0	90	53	0.12	0.732	38	108	5.04	0.025
	Yes	149	48.7	96	52			23	126		
	Missing	7	2.3								
People can take a simple test to find out whether they have HIV.	No	64	20.9	48	13	7.69	0.006	19	44	4.24	0.039
	Yes	237	77.5	138	94			43	192		
	Missing	5	1.6								
A person should know whether or not their sexual partner is HIV positive before agreeing to have sex with them.	No	46	15.0	27	159	0.01	0.932	21	23	23.07	<0.001
	Yes	254	83.0	159	91			40	211		
	Missing	6	2.0								
Apart from HIV/AIDS, there are other diseases that men and women can catch by having sexual intercourse. Have you heard of any of these diseases?	No	28	9.2	18	8	0.32	0.572	17	11	29.20	<0.001
	Yes	273	89.2	170	97			46	225		
	Missing	5	1.6								
Have you ever been tested for HIV/AIDS?	No	201	65.7	133	62	4.42	0.036	46	154	2.16	0.142
	Yes	98	32.0	54	43			15	81		
	Missing	7	2.3								
Do you know your HIV status?	No	213	69.6	140	67	3.01	0.083	53	159	6.56	0.010
	Yes	87	28.4	49	37			10	76		
	Missing	6	2.0								

**Table 4:** Distribution of respondents by condom knowledge and self-efficacy according to reported knowledge of correct condom use and perceived ability to insist on condom use (*N* = 306).

Measure	Value	*n*	%	By correctly				By insist			
				No	Yes	X^2^	*p*	No	Yes	X^2^	*p*
Condoms are an effective way to prevent pregnancy.	No	98	32.0	75	19	15.39	<0.001	30	66	8.94	0.003
	Yes	206	67.3	113	88			33	171		
	Missing	2	0.7								
Girls can suggest using condoms.	No	43	14.1	30	11	2.03	0.154	22	20	31.03	<0.001
	Yes	256	83.7	154	96			38	215		
	Missing	7	2.3								
Do you know any place where young people can obtain condoms?	No	92	30.1	68	19	10.71	0.001	34	57	19.27	<0.001
	Yes	203	66.3	116	84			29	171		
	Missing	11	3.6								
If a girl suggested using condoms to her boyfriend, it would mean that she did not trust him.	No	101	33.0	56	38	1.20	0.273	32	67	11.94	0.001
	Yes	200	65.4	131	67			30	169		
	Missing	5	1.6								
Condoms are an effective way of protecting against sexually transmitted diseases.	No	77	25.2	56	18	6.02	0.014	31	45	24.52	<0.001
	Yes	223	72.9	131	88			31	190		
	Missing	6	2.0								
It is all right for boys and girls to have sex with each other provided that they use a condom.	No	125	40.9	95	26	19.13	<0.001	44	81	25.01	<0.001
	Yes	171	55.9	91	79			19	152		
	Missing	10	3.3								
Most of my friends who have sex with someone use condoms.	No	47	15.4	34	12	19.20	<0.001	17	30	16.56	<0.001
	Yes	91	29.7	41	50			7	83		
	DK	159	52.0	110	45			35	122		
	Missing	9	2.9								
Would you be able to use a condom every time you had sexual intercourse?	No	35	11.4	29	2	13.75	<0.001	21	12	59.72	<0.001
	Yes	237	77.5	139	95			24	213		
	Missing	34	11.1								
Would you be able to use a condom during sex after you had been drinking or taking drugs?	No	215	70.3	145	65	9.40	0.002	53	161	8.61	0.003
	Yes	86	28.1	42	42			8	76		
	Missing	5	1.6								
Would you be able to refuse sex if your partner refuses to use a condom?	No	58	19.0	35	21	0.05	0.819	24	34	19.34	<0.001
	Yes	241	78.7	152	85			37	203		
	Missing	7	2.3								
Would you be able to talk about using condoms with your partners?	No	36	11.8	29	4	9.64	0.002	25	10	62.96	<0.001
	Yes	264	86.3	156	103			36	226		
	Missing	6	2.0								
I would feel embarrassed to put a condom on a partner.	No	130	42.5	87	38	3.02	0.082	40	88	16.02	<0.001
	Yes	170	55.6	101	68			21	149		
	Missing	6	2.0								
I feel I can insist on condoms every time I have sexual intercourse.	No	63	20.6	46	13	6.29	0.012				
	Yes	237	77.5	142	93						
	Missing	6	2.0								
I feel I know how to use a condom correctly	No	189	61.8					46	142	6.29	0.012
	Yes	107	35.0					13	93		
	Missing	10	3.3								

DK = Does not know.

**Table 5:** Distribution of respondent agreement with relationship norms according to knowledge of correct condom use and reported ability to insist on condom use (*N* = 306).

Measure	Value	*n*	%	By correctly				By insist			
				No	Yes	X^2^	*p*	No	Yes	X^2^	*p*
I believe there is nothing wrong with unmarried boys and girls having sexual intercourse if they love each other.	Disagree/Does not know	191	62.4	129	55	8.61	0.003	42	148	0.38	0.537
	Agree	112	36.6	59	52			21	89		
	Missing	3	1.0								
I think sometimes a boy has to force a girl to have sex if he loves her.	Disagree/Does not know	254	83.0	166	85	4.22	0.040	49	204	0.88	0.349
	Agree	46	15.0	22	22			11	32		
	Missing	6	2.0								
A boy will not respect a girl who agrees to have sex with him.	Disagree/Does not know	227	74.2	152	37	5.81	0.016	52	175	2.56	0.110
	Agree	72	23.5	72	34			10	61		
	Missing	7	2.3								
It is sometimes justifiable for a boy to hit his girlfriend.	Disagree/Does not know	206	67.3	130	72	0.21	0.643	44	159	0.39	0.530
	Agree	93	30.4	56	35			17	75		
	Missing	7	2.3								
It is all right for boys and girls to have sex provided they use a condom.	Disagree/Does not know	125	40.9	95	26	19.13	<0.001	44	81	25.01	<0.001
	Agree	171	55.9	91	79			19	152		
	Missing	10	3.3								
Men need sex more frequently than women do.	Disagree/Does not know	146	47.7	105	37	10.72	0.001	32	111	0.49	0.484
	Agree	152	49.7	84	67			29	123		
	Missing	8	2.6								
Men have a right to have sex with their girlfriend whenever they wish.	Disagree/Does not know	239	78.1	155	78	3.24	0.072	46	191	2.08	0.149
	Agree	61	19.9	33	28			17	44		
	Missing	6	2.0								
If a girl has accepted cash or gifts from a man, she must have sex with him.	Disagree/Does not know	227	74.2	148	77	1.87	0.172	36	190	3.85	0.050
	Agree	44	14.4	23	19			12	30		
	Missing	35	11.4								
If your family tells you that you must have sex with someone to help the family, you must do it even if you do not want to do so.	Disagree/Does not know	281	91.8	173	103	1.66	0.197	56	6	2.83	0.093
	Agree	19	6.2	14	4			6	10		
	Missing	6	2.0								

### 3.3 Multivariate analyses

#### 3.3.1 Self-reported knowledge about correct condom use

Results from the logistic regression model of *correctly* are reported in Table 6. Girls who reported that they had not had sexual intercourse had 80% lower odds (AOR 0.19, 95% CI [0.07, 0.54], *p* = 0.002) of reporting they knew how to use condoms correctly compared to their sexually experienced peers. Girls who knew that there was no cure for AIDS had 2.8 times greater odds of reporting that they knew how to use condoms correctly compared to girls who thought that AIDS could be cured or did not know the answer (AOR 2.81, 95% CI;1.23, 6.43], *p* = 0.014). Girls who did not know where to acquire condoms were less likely to report they knew how to use them correctly than girls familiar with sources for them (AOR 0.41, CI 95% [0.18, 0.92], *p* = 0.032). Respondents who disagreed or did not know if it was acceptable for boys and girls to have sex provided they used condoms had lower odds of reporting knowledge of correct condom use compared to girls who thought sex was permissible (AOR 0.42, CI 95% [0.20, 0.91], *p* = 0.028). None of the condom-use self-efficacy or relationship statements that were significant at the bivariate level remained significant in the multivariate model. The model explained 25% of the observed variability.

**Table 6:** Attributes and respondent self-reported condom knowledge and self-efficacy associated with perceived ability to use condoms correctly (*n* = 241).

Measure	Value	OR	*p*	CI95%
Form (*ref.* Form 1)	Form 2	3.21	0.048	1.01, 10.21
	Form 3	1.50	0.513	0.45, 5.0
	Form 4	0.86	0.8	0.22, 3.46
	Form 5	0.53	0.521	0.08, 3.66
Has ever had sexual intercourse (*ref.* yes)	No	0.19	0.002	0.07, 0.54
It is possible to cure AIDS (*ref*. agrees)	Disagrees or does not know	2.81	0.014	1.23, 6.43
Have you ever been tested for HIV (*ref.* yes)	No	0.54	0.078	0.27, 1.07
Condoms are an effective way to prevent pregnancy (*ref.* agrees)	Disagrees or does not know	0.51	0.114	0.22, 1.18
Do you know any place where young people can obtain condoms? (*ref.* yes)	No or does not know	0.41	0.032	0.18, 0.92
Condoms are an effective way to protect against sexually transmitted diseases. (*ref.* agrees)	Disagrees or does not know	1.24	0.661	0.47, 3.30
Would you be able to use a condom every time you had sexual intercourse? (*ref.* yes)	No or does not know	0.28	0.128	0.05, 1.45
Would you be able to use a condom during sex after you had been drinking or taking drugs? (*ref.* yes)	No or does not know	0.82	0.574	0.40, 1.65
Would you be able to talk about using condoms with your partners? (*ref.* yes)	No or does not know	0.26	0.239	0.03, 2.44
Most of my friends who have sex with someone use condoms. (*ref*. agrees)	Disagrees or does not know	0.75	0.456	0.36, 1.59
Men need sex more frequently than women do (*ref.* agrees)	Disagrees or does not know	0.69	0.283	0.35, 1.36
It is all right for boys and girls to have sex provided they use condoms (*ref.* agree)	Disagrees or does not know	0.42	0.028	0.20, 0.91
A boy will not respect a girl who agrees to have sex with him. (*ref.* agrees)	Disagrees or does not know	0.47	0.062	0.21, 1.04
I think sometimes a boy has to force a girl to have sex if he loves hers. (*ref*. agrees)	Disagrees or does not know	0.65	0.38	0.25, 1.70
I believe there is nothing wrong with unmarried boys an girls having sexual intercourse if they love each other.	Disagrees or does not know	0.86	0.66	0.45, 1.72
McFadden’s Pseudo R-Square	0.25			

*Includes only those observations for which there are responses for all variables included in the model.

#### 3.3.2 Perceived ability to insist on condom use

Results from the logistic regression model for *insist* are reported in Table 7. Respondents who did not know their HIV status had 83% lower odds of reporting they felt they could insist on condoms than their peers who knew their status (AOR 0.17, CI 95% [0.04, 0.74], *p* = 0.018). Girls who disagreed or were unsure about the acceptability of their suggesting condoms to their partners had lower odds of feeling they were able to insist on condoms every time (AOR 0.17, CI 95% [0.04, 0.69], *p* = 0.014). Relatedly, girls who reported that they could not or were unsure that they could use condoms every time they had sex had 90% lower odds of being confident they could insist on their use. Several of the condom efficacy measures were significant. Adolescents who reported they would not be able to or were unsure if they could talk with their partners about condoms or refuse to have sex if their partner would not use one had 95% lower odds of reporting they would be able to insist on condom use (AOR 0.05, CI 95% [0.01, 0.22], *p* < 0.001). If girls reported they could not or were unsure about their ability to refuse to have sex with a partner who refused to wear a condom, they were roughly 70% less likely to report they could insist on condom use (AOR 0.29, CI 95% [0.03, 0.90], *p* = 0.032). Reported knowledge about how to use a condom correctly, perceived ability to use a condom during sex or after drinking or taking drugs and feeling embarrassed about putting a condom on a partner were not significant correlates of being able to insist on condoms when controlling for covariates. The McFadden’s Pseudo R-Square for the model was 0.42.

**Table 7:** Attributes and respondent self-reported condom knowledge and efficacy associated with perceived ability to insist on condom use in every sexual encounter (*n* = 244).

Measure	Value	**OR**	***p***	**CI95%**
Has ever had sexual intercourse (*ref.* yes)	No	0.55	0.449	0.12, 2.60
Water source (ref. private or communal pipe or tap)	River, creek, stream, other	5.02	0.294	0.25, 101.78
It is possible to cure AIDS (*ref*. agrees)	Disagrees or does not know	1.99	0.241	0.63, 6.29
Do you know your HIV status? (*ref.* yes)	No	0.17	0.018	0.04, 0.74
A person should know whether their sexual partner is HIV positive before agreeing to have sex with them. (*ref.* agrees)	Disagrees or does not know	0.50	0.279	0.15, 1.75
People can take a simple test to find out if they have HIV (ref. agrees)	Disagrees or does not know	0.60	0.451	0.16, 2.27
A person with HIV always looks emaciated or unhealthy in some way. (*ref.* agrees)	Disagrees or does not know	0.58	0.339	0.19, 1.77
Condoms are an effective way to prevent pregnancy (*ref.* agrees)	Disagrees or does not know	1.38	0.63	0.38, 5.04
Do you know where young people can obtain condoms? (*ref.* yes)	No or does not know	0.68	0.482	0.23, 2.01
Condoms are an effective way to protect against sexually transmitted diseases. (*ref.* agrees)	Disagrees or does not know	0.49	0.259	0.14, 1.70
If a girl suggested using condoms to her boyfriend, it would mean that she did not trust him. (*ref.* agrees)	Disagrees or does not know	1.71	0.379	0.52, 5.67
Girls can suggest using condoms. (*ref.* agrees)	Disagrees or does not know	0.01	0.014	0.04, 0.69
Would you be able to use a condom every time you had sexual intercourse? (*ref.* yes)	No or does not know	0.10	0.001	0.02, 0.38
Would you be able to use a condom during sex after you had been drinking or taking drugs? (*ref.* yes)	No or does not know	0.81	0.734	0.25, 2.68
Would you be able to talk about using condoms with your partners? (*ref.* yes)	No or does not know	0.05	<0.001	0.01, 0.22
I know how to use a condom correctly. (*ref.* agrees)	No or does not know	2.05	0.231	0.63, 6.62
I would feel embarrassed to put a condom on my partner (*ref.* agrees)	Disagrees or does not know	0.96	0.943	0.33, 2.83
Would you be able to refuse sex if your partner refuses to use a condom? (*ref.* yes)	No or does not know	0.29	0.032	0.10, 0.90
McFadden’s pseudo *R*-square		0.42		

*Includes only those observations for which there were responses for all variables included in the model.

## 4 Discussion

Multiple risk factors have been identified in both academic and mainstream literature that increase the vulnerability of adolescents to condomless sex and risk of HIV. The extent to which certain of these factors predominate as correlates of sexual risk-taking, however, varies by b setting, age, gender, and the methods by which condom use, knowledge, and efficacy are operationalized, making cross-study comparisons difficult to interpret. For example, this study identified a positive correlation between various measures of condom self-efficacy and the intention to use condoms consistently; similar results have been reported in a number of studies in other countries in SSA (18, 21, 22, 24, 38–42) but, of these, one examined university students only (21) while one used a sample of 15–24 years olds in which findings were not disaggregated by age (39). Three studies restricted their samples to sexually active young people (21, 22, 24). Numerous studies looked exclusively at secondary school adolescents (18, 22, 24, 38, 40–42); importantly, however, given the focus of this study on adolescent girls, only two studies of secondary school students presented findings that were disaggregated by gender (22, 38) and one of these exclusively sampled sexually active adolescents (22). Of the Botswana studies that found a positive correlation between perceived self-efficacy and condom use intention among adolescents (43–45) only one study reported findings disaggregated by gender (43). The paucity of data specific to adolescent girls is surprising given the sizeable body of evidence that adolescent girls and young women are at disproportionate risk of HIV infection compared to males (14).

Only three measures of perceived ability – condom use every time, communication with partner about condoms, and refusal to have sex if one’s partner refused to use a condom – were significant in the multivariate model assessing the perceived ability to insist on condom use every time. Communication with one’s partner about HIV and condoms has been shown to be a significant predictor of condom use in previous studies examining adolescents and young adults in SSA (46, 47). Analysis of responses from 12 to 18 adolescents found that sexual abstainers where more confident that sexually active youth in their ability to avoid or refuse sex (39). In this study, reported sexual experience was not a significant factor with self-reported ability to insist on condoms in the bivariate models but was significant in the multivariate model assessing perceived knowledge of correct condom use.

While it may seem logical that adolescents who have not had sexual intercourse or do not know where they can acquire condoms are more likely to report that they do not know how to use a condom correctly, other findings in the model may warrant further explanation. In this study, girls in the lowest grade (Form 2) and presumably among the youngest and least sexually experienced students surveyed, had significantly greater odds of reporting they were knowledgeable about correct condom use. Messaging about condoms is ubiquitous in Botswana and most programs targeting risk-reduction in adolescents include demonstrations and discussions about correct condom usage. Young and inexperienced girls may naively think that they have the requisite knowledge but have not actually tested this in real life. Not surprisingly girls who do not believe or question whether it is all right for boys and girls to have sex provided they use condoms have lower odds of reporting knowledge of correct condom use compared to girls who perceive that sex at their age is acceptable.

Findings in the *insist* model suggest that girls who do not know their HIV status have lower odds of perceived ability to insist on the use of condoms compared to girls who do. A 2017 systematic review of sexual risk-taking among HIV-positive youth in SSA similarly reported that knowledge of one’s HIV status was positively associated with reduced unprotected sex among adolescents. Findings from a study of male and female adolescents in Ghana found that the intention to use condoms was positively associated with recognition that condoms would prevent HIV-transmission (48). Adolescents who know their status have visited testing and/or treatment centers where the importance of consistent use of condoms is heavily emphasized. Adolescents who have not been tested may not ‘connect the dots’ between HIV transmission and unprotected sex to the same extent as their status-aware peers.

Most findings as to why adolescent girls continue to be at the epicenter of new cases of HIV in Sub-Saharan Africa are based on analyses that do not disaggregate the youngest or sexually naïve girls from similarly aged males or older and more sexually experienced females. By focusing exclusively on 14–19-year-old adolescent girls, this study offers the opportunity to identify potential points of intervention that might strengthen the intention, capacity, and confidence of sexually experienced girls to insist on and correctly use condoms in their next and future sexual encounters. For obvious reasons, much of the scholarly literature on HIV risk and condom use centers on the reported behaviors of girls who have already had sexual intercourse, i.e., the use of condoms every time they have had sex, or at the time of first or most recent sexual intercourse. Many sexually active adolescents, both males and females, have already engaged in high-risk behaviors, as evidenced by the high rates of non-condom use among young people in SSA reported in these studies (5, 49, 50). Interventions directed at these young people must focus on sexual behavior change and their effectiveness measured by the extent that non-condom users will report consistent and correct condom users in the future.

Arguably, HIV-risk reduction programs aimed at sexually naïve adolescents have a different goal, that of building and strengthening intentions to use condoms consistently and correctly from their very first sexual encounter *before* a risk trajectory becomes entrenched. Eighty-four percent of the adolescent girls participating in this study reported that they had never had sexual intercourse, a finding that aligns with previous research among similarly aged cohorts in Botswana (17, 44). A substantial body of research supports the idea that intention to perform a behavior is strongly linked to the actual performance of that behavior (51, 52). Over the course of the past two decades, the theory of planned behavior (53) has been used to predict condom use among adolescents in SSA (41, 42, 48, 54–56). Previous research among secondary school adolescents in Botswana has also shown that the theory of planned behavior is strongly predictive of condom use intention in this age group (44). According to the theory, the stronger the intentions to use condoms, the more likely it is that condom-use behavior will follow (43, 44). Intention is shaped by three critical determinants: attitude (the degree to which an individual has a favorable understanding of the behavior), subjective norms (perceived social pressures to engage in the behavior), and perceived behavioral control (an individual belief in how easy or difficult a behavior may be to perform). Findings from this study suggest that most adolescent girls have a positive attitude towards condom use, with most respondents believing that it is all right for boys and girls to have sex with each other provided they use condoms. Interestingly, a study of 12–18 year old males and females in Uganda found that sexual abstainers were more likely than sexually active youth to have positive perceptions about condom use and be confident in their ability to avoid or refuse sex (40). Interventions that build girls’ confidence in communicating about sex, their knowledge about correct condom use, and their intentions to insist on condoms could equip girls with the confidence to refuse condomless sex at that first encounter and to feel positive about condom use. Based on findings from this study, WAR, our collaborating partner, has initiated new programming for adolescent girls in schools that incorporate activities emphasizing life skills, decision-making, bodily autonomy, communication, and confidence, into the nationally mandated comprehensive sexuality education program for all adolescents. Programs such as DREAMS, which has been shown to increase HIV knowledge, condom use, and HIV testing, might be particularly useful in this setting (57, 58).

One often-discussed impediment to the translation of condom use intention into condom use behavior by adolescent girls in this setting lies in their perception of male power in relationships. While not majority views, a noticeable proportion of adolescent girls think boys have to sometimes force girls to have sex (15%), will not respect girls who have sex (23.5%), can be justified in hitting their girlfriends (30.4%), need sex more than girls do (49.7%), and have a right to have sex with their girlfriends whenever they wish (19.9%). While a number of these normative statements were significant at the bivariate level, none of these factors remained significant in the multivariate models that controlled for other covariates. Nonetheless, interventions to strengthen intentions should include efforts to shift how adolescent girls view these patriarchal beliefs, as it has been reported that enhancing gender equity norms at the peer level may have protective effects (18, 59).

### 4.1 Implications for policy and practice

Findings from this analysis indicate that interventions that focus on improving HIV knowledge among adolescent girls would be highly beneficial. Even though awareness of HIV risk is high in this population, misperceptions about HIV persist in 20–25% of these adolescent girls and were predictive of low perceived ability to use condoms correctly. Iterative messaging from teachers and health professionals in secondary school settings about HIV facts could be easily reinforced in comprehensive sexuality education programs mandated by the Botswana government. More intractable to change may be the endorsement by adolescent girls of norms that defer to males in sexual relations, although activities that encourage girls to talk about sexual matters with boys are likely to enhance their ability to communicate with their partners over condom use.

All schools in Botswana at present are required to offer comprehensive sexuality education (CSE) across all grades (60), although the quality of these life-skill building programs suffer in implementation because teachers and counsellors lack specific training in talking with students about the cognitive, emotional, physical, and social aspects of sexuality. There is strong evidence that schools are a critical venue for outreach to adolescents about HIV prevention and sexual behaviors (61). In the Northwest District, CSE is scheduled during open periods that are often used for other purposes. CSE needs to be recentered within Botswana’s secondary school curricula and more skills-building opportunities for teachers and counsellors provided.

## 5 Limitations

This study has several limitations. As the data used for analysis were cross-sectional, one cannot draw definitive conclusions about the temporal or causal relationships among variables. Data were self-reported by respondents and may well reflect under- and over-reporting of feelings and behaviors. Respondents may have been influenced by social desirability to provide answers that seemed acceptable or expected rather than to report their true feelings. Importantly, responses may reflect perceived condom knowledge and negotiation skill rather than actual knowledge or ability.

Study participants at each school were recruited from a purposive sample of students attending a life-skills-building session. The timing of these sessions varied and not all students in a school participated at the same time. This may have resulted in a cluster effect within an individual school in which the students in a particular session seem to have more traits in common because of their form/grade than they do with students in other forms within the same school. Along the same line, we have not used cluster sampling to account for the possibility that students in a particular school may have more in common with each other than with students at other locations.

Pursuing a goal of parsimony in the number of variables in the multivariate analyses, we included only those variables shown in the bivariate models to be statistically significant at *p* < 0.05; there is a risk that this variable selection method may have resulted in the omission of some factors that contribute to the outcome variables under study.

Lastly, this study contributes to an understanding of how adolescent girls in one district in Botswana perceive their abilities to use condoms correctly and consistently in their sexual encounters. These findings may serve as a valuable resource for regional education authorities, teachers, schools, and non-governmental organizations in the Northwest District where this study took place. The extent to which the findings are generalizable to other districts in Botswana or other parts of Sub-Saharan Africa, however, needs to be viewed with caution and would merit further validation in those settings.

## 6 Conclusion

Adolescent girls in Botswana continue to bear a disproportionate burden of HIV incidence. Interventions targeting adolescent girls that focus on improving knowledge about HIV and correct condom use, enhance capacity to insist on condom use and encourage communication with sexual partners about condoms may help this vulnerable population translate their intentions to use condoms correctly and consistently into actual behavior. In particular, programmatic initiatives designed to enhance condom self-efficacy among sexually uninitiated girls could encourage the correct and consistent use of condoms from the first sexual encounter and should be supported.

## Data Availability

The raw data supporting the conclusions of this article will be made available by the authors, without undue reservation.

## Footnotes

1
https://www.qualtrics.com
